# Supplementary material for: Extracellular Delivery of Functional Mitochondria Rescues the Dysfunction of CD4+ T Cells in Aging
Source: Adv Sci (Weinh). 2023 Nov 21;11(5):2303664. doi: 10.1002/advs.202303664 (PMC10837346; doi:10.1002/advs.202303664)
Supplement: Supplementary file 1 — Supporting Information [file ADVS-11-2303664-s001.pdf]

ADVANCED SCIENCE
Open Access

Supporting Information

for *Adv. Sci.*, DOI 10.1002/advs.202303664

Extracellular Delivery of Functional Mitochondria Rescues the Dysfunction of $CD4^+$ T Cells in Aging

Colwyn A. Headley, Shalini Gautam, Angelica Olmo-Fontanez, Andreu Garcia-Vilanova, Varun Dwivedi, Anwari Akhter, Alyssa Schami, Kevin Chiem, Russell Ault, Hao Zhang, Hong Cai, Alison Whigham, Jennifer Delgado, Amberlee Hicks, Philip S. Tsao, Jonathan Gelfond, Luis Martinez-Sobrido, Yufeng Wang, Jordi B. Torrelles and Joanne Turner**

Supplementary figures.

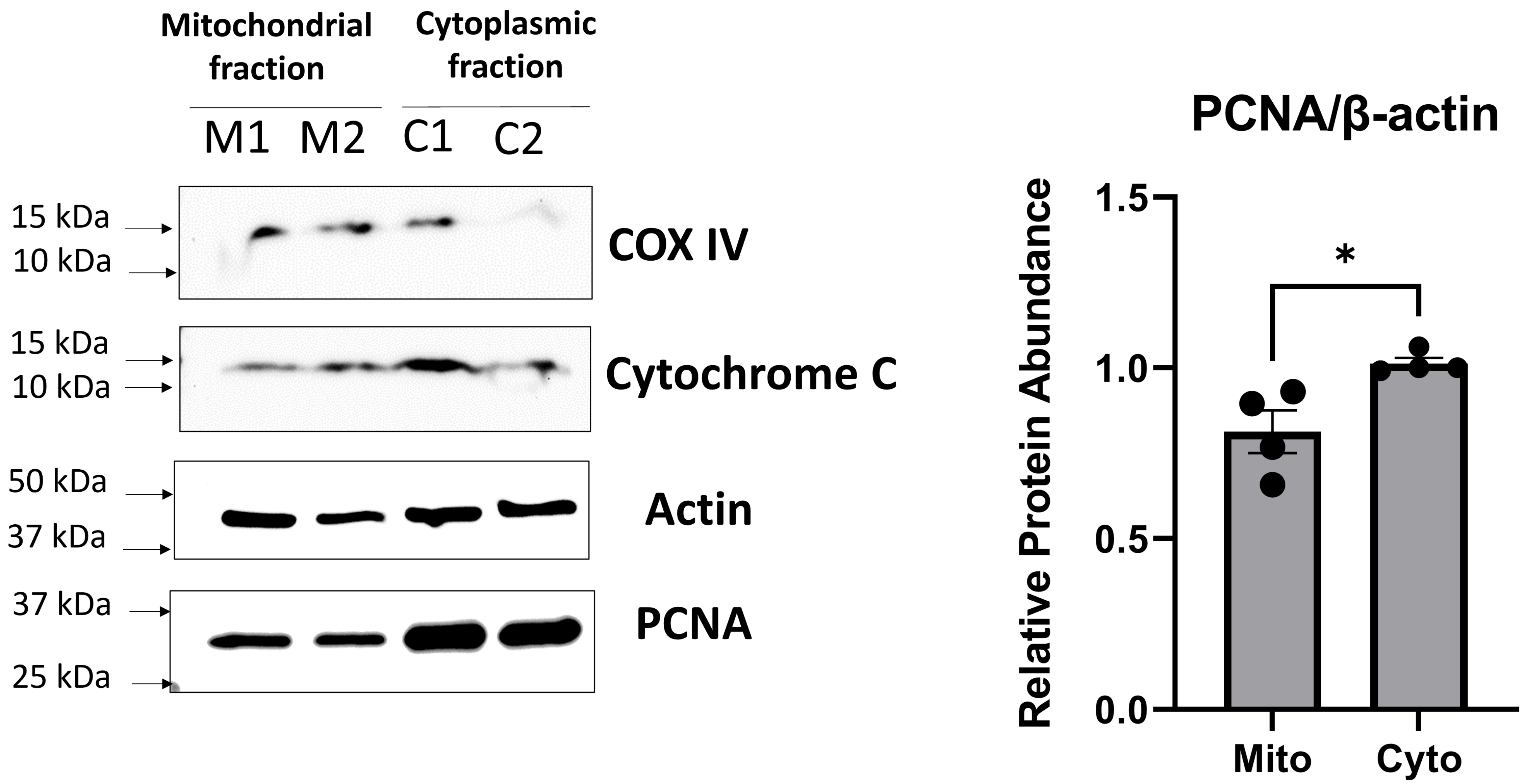
Mitochondrial fraction
Cytoplasmic fraction
M1
M2
C1
C2
15 kDa
10 kDa
COX IV
15 kDa
10 kDa
Cytochrome C
50 kDa
37 kDa
Actin
37 kDa
25 kDa
PCNA
PCNA/β-actin
Relative Protein Abundance
1.5
1.0
0.5
0.0
*
Mito
Cyto

Supp. Fig. 1. Purity of mitochondrial isolation. A) Representative western blot images of probed mitochondrial and nuclear proteins in the mitochondrial fraction and cell fragments/ unlysed cells after mitochondrial isolation. **B)** Relative amount of PCNA normalized to B-actin. Western Blot images were analyzed using high contrast settings. (n=4), $p<0.05$=significant (*) using unpaired Student's t-test.

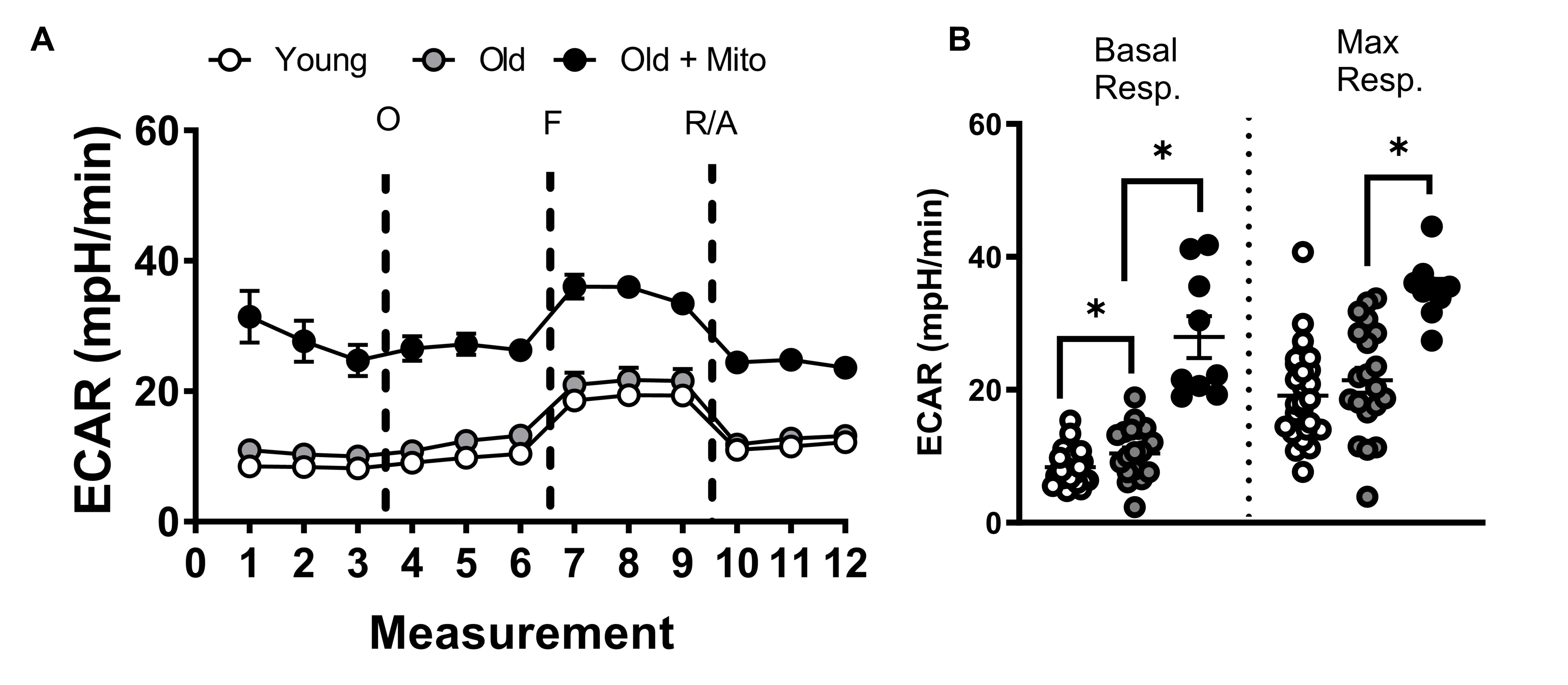

A
Young
Old
Old + Mito
ECAR (mpH/min)
O
F
R/A
60
40
20
0
0 1 2 3 4 5 6 7 8 9 10 11 12
Measurement
B
Basal Resp.
Max Resp.
ECAR (mpH/min)
60
40
20
0
*
*
*

Supp. Fig. 2. Mito-transfer increases ECAR of CD4$^+$ T cells from old mice. A) Kinetic curve of ECAR collected from mito-stress test assay, **B)** ECAR corresponding to basal and maximal respiration of CD4$^+$ T cells. 3-4 mice were used per group and experiments were replicated twice (n=3), $p<0.05$=significant (*) using unpaired Student's t-test.

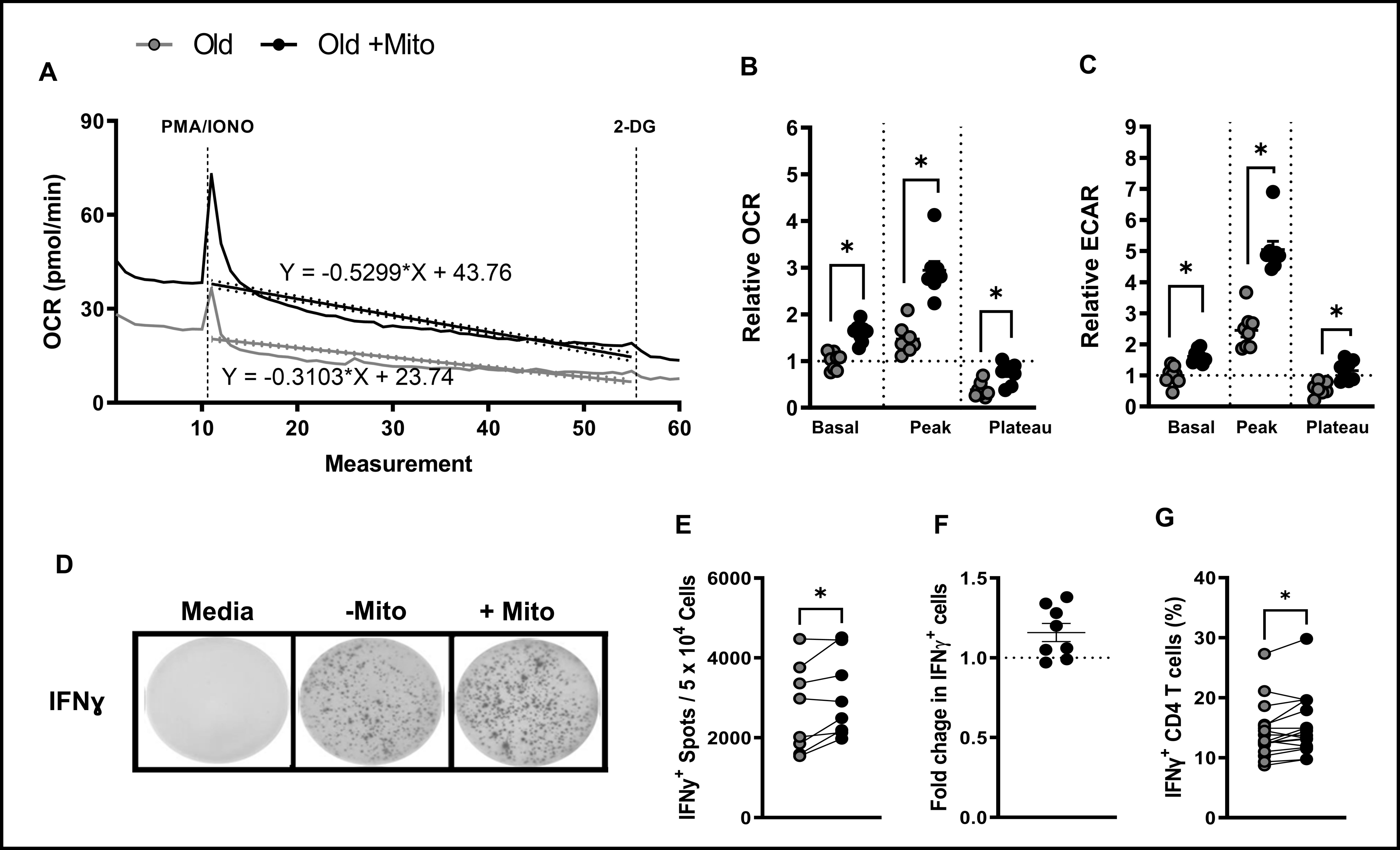
Old
Old +Mito
A
PMA/IONO
2-DG
OCR (pmol/min)
Y = -0.5299*X + 43.76
Y = -0.3103*X + 23.74
Measurement
B
Relative OCR
Basal
Peak
Plateau
C
Relative ECAR
Basal
Peak
Plateau
D
Media
-Mito
+ Mito
IFNγ
E
IFNγ+ Spots / 5 x 10^4 Cells
F
Fold chage in IFNγ+ cells
G
IFNγ+ CD4 T cells (%)

Supp. Fig. 3. Mito-transfer enhaced T cell activation induced metabolic switch and increased IFNγ^{+} CD4^{+} T cells. A) The slope of OCR depression after acute injection of PMA/IONO in mito-transferred and non-manipulated CD4+ T cells from old mice. The relative change in **B)** OCR and **C)** ECAR of activated T cells (non-normalized to group). **D-G)** CD4^{+} T cells from old mice with or without mito-transfer were stimulated with PMA/Ionomycin, after which various assays were used to quantify IFNγ production. **D)** Representative EliSpot images, **E)** the number IFNγ^{+} producing T cells, and **F)** the fold change in IFNγ^{+} producing CD4^{+} T cells from old mice with or without mito-transfer, after stimulation with PMA/Ionomycin (24hr). The **G)** percent of IFNɣ$^{+}$ CD4^{+} T cells via intracellular cytokine staining, in CD4^{+} T cells from old mice with or without mito-transfer, after stimulation with PMA/Ionomycin (4h). 3-4 mice per group, repeated at least once ($n \geq 2$). $p < 0.05$ = significant (*) using paired Student's *t*-test.

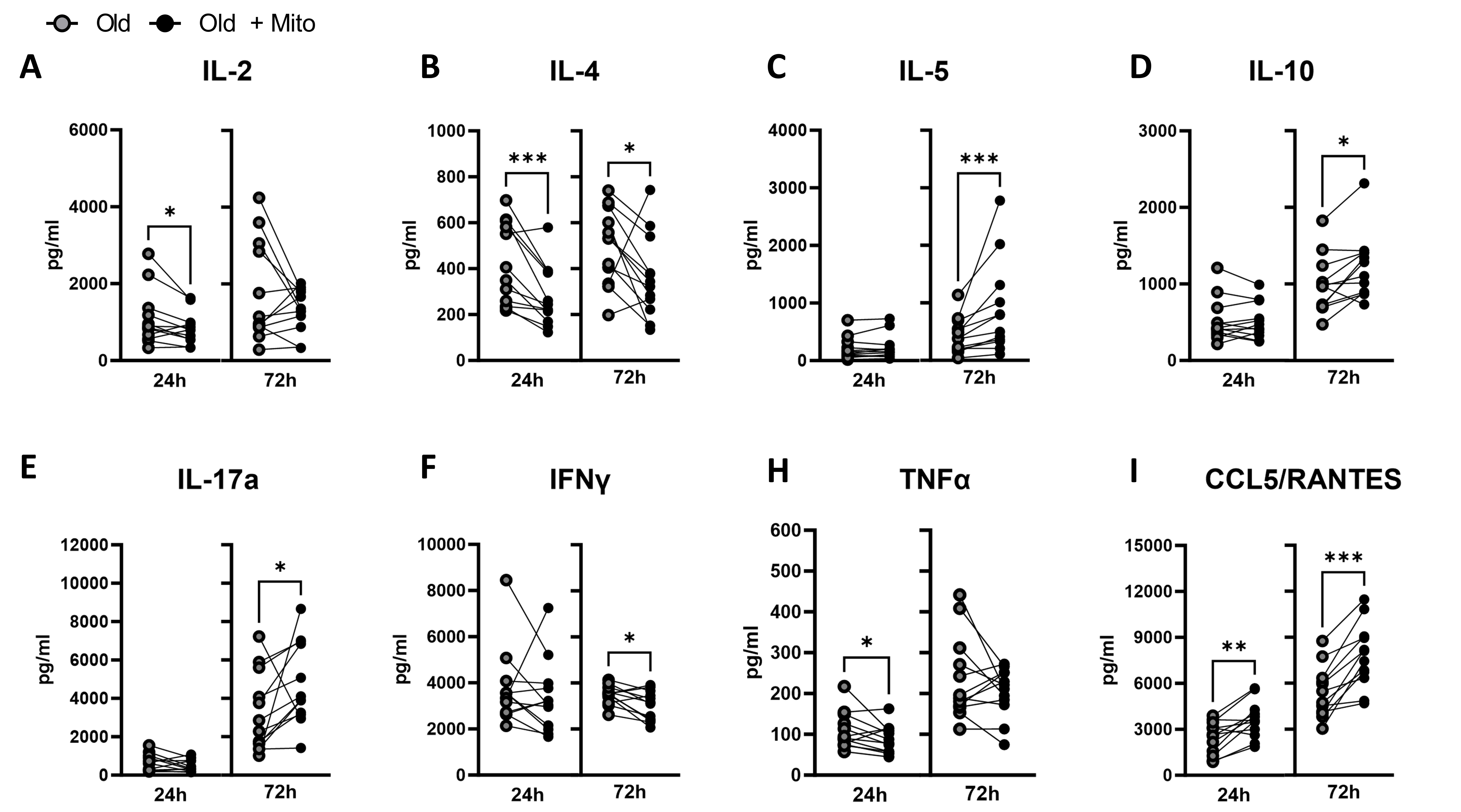

Old
Old + Mito
A
IL-2
B
IL-4
C
IL-5
D
IL-10
E
IL-17a
F
IFNγ
H
TNFα
I
CCL5/RANTES
pg/ml
24h
72h

Supp. Fig. 4. Mito-transfer alters T cell activation & cytokine production of $CD4^+$ T cells in old mice. $CD4^+$ T cells from old mice with or without mito-transfer were stimulated with PMA/Ionomycin. After 24 and 72 h of stimulation with PMA/Ionomycin, the supernatants were examined by Luminex array for cytokines produced. $p \leq 0.05$ = *, $p \leq 0.01$ = **, or $p \leq 0.001$ = *** using paired Student's *t*-test.

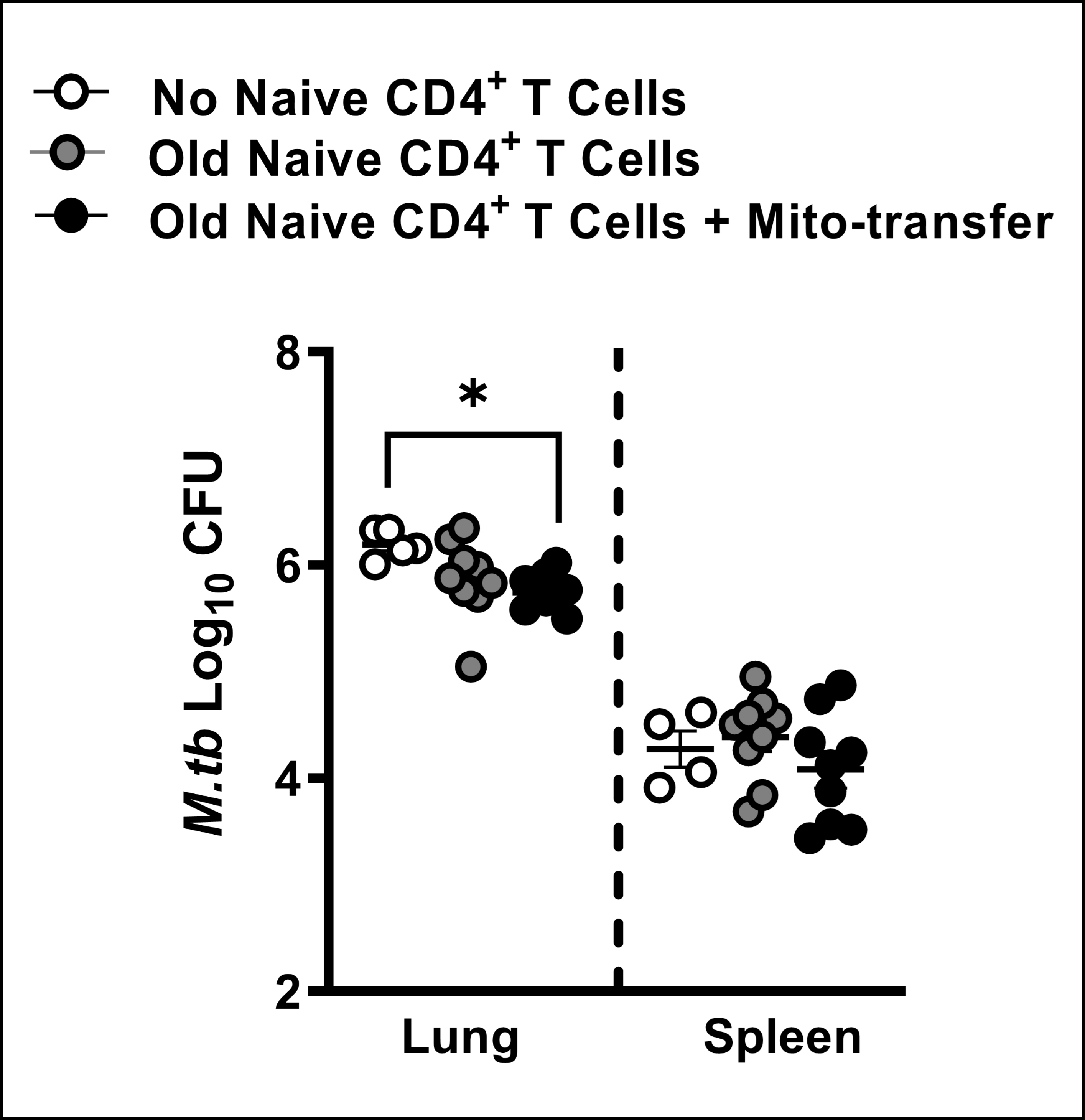

No Naive CD4+ T Cells
Old Naive CD4+ T Cells
Old Naive CD4+ T Cells + Mito-transfer
M.tb Log10 CFU
8
6
4
2
*
Lung
Spleen

Supp. Fig. 5. Mito-transfer in naïve CD4 T cells from old mice protected mice against pathogens. Naïve $CD4^+$ T cells from old mice with or without mito-transfer, or PBS were tail vein injected in Rag1-KO mice that were subsequently infected with *M.tb*. Lung and spleen CFU burden after 21 days of *M.tb* infection in Rag1-KO mice adoptively treated with 1x 10^6 naïve $CD4^+$ T cells from old mice with or without mito-transfer. 4-8 mice per group, with $p \leq 0.05$ = *, using unpaired Student's *t*-test.

Uniprot ID	Gene ID	P.Val	FDR	O1	O2	O3	OM1	OM2	OM3
P24270	Cat	0.046	0.148	0.177	0.000	-0.253	1.040	0.403	0.494
Q99LX0	Park7	0.001	0.015	0.016	-0.257	0.000	-1.140	-1.450	-1.350
P11247	Mpo	0.459	0.719	1.480	0.000	-0.328	0.674	-0.715	-0.546
Q9JMH6	Txnrd1	0.030	0.114	0.000	-0.115	0.093	-0.408	-1.270	-1.320
P08228	Sod1	0.000	0.013	-0.224	0.010	0.000	-1.080	-1.290	-1.170
Q9ERR7	Sep15	0.054	0.164	0.000	0.000	0.112	0.582	0.658	0.160
O08709	Prdx6	0.003	0.031	0.085	0.000	-0.156	-1.090	-1.810	-1.670
P19157	Gstp1	0.004	0.036	-0.138	0.000	0.106	-1.050	-1.380	-0.809
Q8CDN6	Txnl1	0.008	0.051	0.000	0.040	-0.283	-0.969	-1.380	-0.821
P11352	Gpx1	0.131	0.294	0.457	0.000	-0.235	-0.028	-0.804	-0.877
P99029	Prdx5	0.996	1.000	0.234	0.000	-0.266	0.051	-0.201	0.121
Q61171	Prdx2	0.040	0.134	0.089	0.000	-0.208	-0.353	-0.755	-0.402
P09671	Sod2	0.023	0.096	-0.061	0.348	0.000	0.715	0.476	0.682
P02088	Hbb-b1	0.050	0.155	0.499	0.000	-0.660	-1.080	-1.670	-3.060
P35700	Prdx1	0.130	0.293	0.028	0.000	-0.030	0.421	-0.006	0.325
Q99LJ6	Gpx7	0.000	0.012	0.150	-1.130	0.000	5.360	4.910	5.340
Q9D7B7	Gpx8	0.000	0.007	0.000	0.540	-0.171	5.920	5.450	5.940
P31725	S100a9	0.374	0.622	1.930	0.000	-0.136	0.985	-2.240	-0.397
P01942	Hba	0.040	0.135	0.473	0.000	-0.794	-0.999	-1.950	-2.470
P49290	Epx	0.465	0.724	1.320	-0.368	0.000	0.885	-1.770	-0.395
P22437	Ptgs1	0.902	1.000	0.376	0.000	-0.608	0.313	-0.174	-0.239
P62342	Selt	0.115	0.270	0.000	0.100	-0.390	0.284	0.120	0.255
Q91VS7	Mgst1	0.030	0.113	0.999	0.000	-0.245	1.580	1.500	2.410
P47791	Gsr	0.018	0.084	0.341	0.000	-0.118	-0.466	-1.080	-1.080
Q9JLT4	Txnrd2	0.839	1.000	0.000	0.351	-0.041	0.125	0.030	0.247
Q6ZPY7	Kdm3b	0.027	0.107	0.000	0.495	-0.124	-0.530	-1.600	-1.240
Q9CQM5	Txndc17	0.008	0.055	-0.143	0.362	0.000	-1.500	-1.760	-2.830
P08226	Apoe	0.508	0.772	0.861	-0.299	0.000	0.960	0.161	0.360
P20108	Prdx3	0.006	0.046	0.214	-0.058	0.000	0.778	0.509	0.675
Q61646	Hp	0.202	0.399	1.520	-0.351	0.000	0.109	-1.160	-0.961
O08807	Prdx4	0.256	0.470	0.038	0.000	-0.002	0.439	-0.070	0.262
Q9DCM2	Gstk1	0.701	0.977	0.010	0.000	-0.212	0.377	-0.571	-0.378
Q9BCZ4	Vimp	0.732	1.000	-0.386	0.179	0.000	0.206	-0.445	-0.249
Q9WU84	Ccs	0.004	0.036	-0.087	0.000	0.263	-0.735	-1.230	-1.050
P30355	Alox5ap	0.424	0.678	0.786	0.000	-0.301	1.160	0.291	0.218
P02104	Hbb-y	0.451	0.708	0.641	0.000	-2.510	-0.842	-1.560	-2.020
P97346	Nxn	0.333	0.570	0.333	0.000	-1.090	-2.170	0.304	-1.790

Uniprot	Gene ID	P.Val	FDR	O1	O2	O3	OM1	OM2	OM3
P24270	Cat	0.046	0.148	0.177	0.000	-0.253	1.040	0.403	0.494
P47738	Aldh2	0.815	1.000	0.398	0.000	-0.923	0.773	-0.269	-0.602

Q8VDK1	Nit1	0.269	0.488	0.029	0.000	-0.100	-0.074	-0.053	-0.391
P47740	Aldh3a2	0.057	0.169	0.000	0.097	-0.121	0.310	0.102	0.328
Q9JMH6	Txnrd1	0.030	0.114	0.000	-0.115	0.093	-0.408	-1.270	-1.320
P08228	Sod1	0.000	0.013	-0.224	0.010	0.000	-1.080	-1.290	-1.170
Q99J99	Mpst	0.334	0.571	0.163	0.000	-0.160	0.378	-0.932	-0.891
Q9JHW2	Nit2	0.309	0.542	-0.141	0.431	0.000	0.296	0.245	0.362
O08709	Prdx6	0.003	0.031	0.085	0.000	-0.156	-1.090	-1.810	-1.670
P11352	Gpx1	0.131	0.294	0.457	0.000	-0.235	-0.028	-0.804	-0.877
P99029	Prdx5	0.996	1.000	0.234	0.000	-0.266	0.051	-0.201	0.121
Q61171	Prdx2	0.040	0.134	0.089	0.000	-0.208	-0.353	-0.755	-0.402
P09671	Sod2	0.023	0.096	-0.061	0.348	0.000	0.715	0.476	0.682
Q64133	Maoa	0.000	0.011	0.151	0.000	-0.761	3.640	3.580	3.770
Q8R164	Bphl	0.031	0.114	0.197	0.000	-0.133	0.714	0.305	0.778
Q9DBF1	Aldh7a1	0.000	0.009	0.000	0.318	-0.191	2.700	2.460	2.520
Q9WVL0	Gstz1	0.112	0.265	0.000	0.163	-0.024	1.190	0.487	0.239
Q9DCN2	Cyb5r3	0.000	0.005	0.000	0.161	-0.051	2.080	1.950	2.120
Q8BW75	Maob	0.000	0.013	0.667	0.000	-0.370	4.120	3.720	4.010
Q9CQX2	Cyb5b	0.013	0.071	-0.164	0.064	0.000	0.975	0.423	0.947
Q99KB8	Hagh	0.754	1.000	0.142	0.000	-0.442	-0.018	-0.512	-0.014
Q9JLJ2	Aldh9a1	0.081	0.213	0.030	-0.088	0.000	-0.239	-1.410	-0.779
Q91VS7	Mgst1	0.030	0.113	0.999	0.000	-0.245	1.580	1.500	2.410
P47791	Gsr	0.018	0.084	0.341	0.000	-0.118	-0.466	-1.080	-1.080
Q9JLT4	Txnrd2	0.839	1.000	0.000	0.351	-0.041	0.125	0.030	0.247
P20108	Prdx3	0.006	0.046	0.214	-0.058	0.000	0.778	0.509	0.675
O08807	Prdx4	0.256	0.470	0.038	0.000	-0.002	0.439	-0.070	0.262
Q9D6Y7	Msra	0.068	0.189	0.549	-0.217	0.000	-0.252	-0.768	-0.814
Q9DCM2	Gstk1	0.701	0.977	0.010	0.000	-0.212	0.377	-0.571	-0.378

Supp. Table 1. Antioxidant & mitochondrial detoxification proteins were detected in CD4$^+$ T cells isolated from old mice after mito-transfer and in non-manipulated CD4$^+$ T cells from old mice. CD4$^+$ T cells from young and old mice, and from old mice after mito-transfer were cultured for 4 h before processing for mass spectrometry analysis. Data expressed as median protein Log2 fold change of CD4$^+$ T cells from old mice, from 3 individual old mice (paired experiment)

Uniprot ID	Gene ID	P. Val	FDR	O1	O2	O3	OM1	OM2	OM3
Q9JIK9	Mrps34	0.049	0.154	0.000	0.757	-0.497	1.210	1.140	1.010
Q8K1Z0	Coq9	0.032	0.117	0.000	0.186	-0.023	0.862	0.388	0.472
P97450	Atp5j	0.033	0.121	0.000	0.125	-0.630	1.010	0.420	1.140

Q9D0K2	Oxct1	0.000	0.012	0.000	0.092	-0.243	1.760	1.440	1.670
P52825	Cpt2	0.312	0.545	0.000	0.208	-0.370	1.210	0.043	0.051
Q9D6J6	Ndufv2	0.049	0.154	0.000	0.342	-0.183	0.718	0.363	0.856
O08734	Bak1	0.010	0.060	0.000	0.028	-0.547	0.687	0.764	0.655
P24270	Cat	0.046	0.148	0.177	0.000	-0.253	1.040	0.403	0.494
Q8BJZ4	Mrps35	0.007	0.051	0.062	0.000	-0.636	1.340	1.050	0.901
D3Z7P3	Gls	0.500	0.765	0.000	0.006	-0.037	0.732	-0.191	0.043
Q9JI39	Abcb10	0.138	0.303	0.860	-0.266	0.000	1.480	0.456	1.190
Q9CQJ8	Ndufb9	0.003	0.033	-0.196	0.220	0.000	0.740	0.744	0.811
P97742	Cpt1a	0.015	0.076	0.000	0.296	-0.452	0.925	0.756	0.903
P47738	Aldh2	0.815	1.000	0.398	0.000	-0.923	0.773	-0.269	-0.602
Q9Z2I8	Suclg2	0.566	0.835	0.026	0.000	-0.159	0.510	-0.235	0.016
Q9DCX2	Atp5h	0.025	0.101	0.000	0.466	-0.336	1.070	1.120	0.719
Q8VE22	Mrps23	0.006	0.044	-0.054	0.132	0.000	0.889	0.501	0.810
Q9D404	Oxsm	0.917	1.000	0.000	0.611	-0.220	0.234	0.026	0.045
Q99JB2	Stoml2	0.007	0.048	0.000	0.206	-0.196	0.991	0.626	0.828
P59017	Bcl2l13	0.118	0.274	0.000	-0.077	0.194	-0.067	-0.784	-0.362
Q8BGH2	Samm50	0.000	0.013	0.000	0.083	-0.194	0.973	0.871	0.934
Q9CZR8	Tsfm	0.707	0.983	0.245	0.000	-0.207	0.426	-0.475	-0.282
Q6PB66	Lrpprc	0.035	0.125	0.000	0.121	-0.292	0.941	0.428	0.430
O09111	Ndufb11	0.001	0.018	0.019	-0.197	0.000	0.578	0.575	0.648
Q9D6R2	Idh3a	0.546	0.814	0.000	0.280	-0.166	0.748	-0.097	0.040
Q9CQ92	Fis1	0.078	0.209	0.240	-0.067	0.000	-0.226	-0.500	-0.127
Q78PY7	Snd1	0.431	0.687	0.121	0.000	-0.224	0.151	-0.543	-0.309
P29758	Oat	0.001	0.018	0.000	0.170	-0.473	1.810	1.920	2.200
O08749	Dld	0.005	0.039	0.000	0.188	-0.179	0.904	0.625	0.763
P38647	Hspa9	0.072	0.196	0.035	0.000	-0.356	1.510	0.407	0.498
P63038	Hspd1	0.006	0.044	0.000	0.119	-0.240	1.090	0.650	0.822
Q9DBL1	Acadsb	0.001	0.022	0.000	0.020	-0.201	1.980	1.390	1.380
Q9Z2I0	Letm1	0.002	0.025	0.000	0.057	-0.198	0.565	0.495	0.591
Q07813	Bax	0.323	0.559	-0.110	0.000	0.085	0.671	-0.114	0.210
Q99JY0	Hadhb	0.462	0.721	-0.070	0.350	0.000	0.625	-0.024	0.237
Q9Z110	Aldh18a1	0.109	0.261	0.000	0.052	-0.331	1.350	0.197	0.449
Q8VDK1	Nit1	0.269	0.488	0.029	0.000	-0.100	-0.074	-0.053	-0.391
Q9CPY7	Lap3	0.806	1.000	0.158	0.000	-0.383	0.568	-0.516	0.000
Q8BH59	Slc25a12	0.037	0.127	-0.084	0.153	0.000	0.368	0.204	0.480
Q9R0X4	Acot9	0.058	0.171	0.000	0.694	-0.021	1.030	0.770	0.829
Q8BWT1	Acaa2	0.089	0.228	0.000	0.088	-0.212	0.592	0.136	0.245
Q99N84	Mrps18b	0.001	0.019	-0.100	0.262	0.000	1.290	1.020	1.220
Q99LX0	Park7	0.001	0.015	0.016	-0.257	0.000	-1.140	-1.450	-1.350
Q9CQ06	Mrpl24	0.031	0.115	0.158	0.000	-0.175	0.302	0.820	0.865
P47740	Aldh3a2	0.057	0.169	0.000	0.097	-0.121	0.310	0.102	0.328
Q9CPV4	Glod4	0.023	0.096	0.000	0.150	-0.049	-0.326	-0.824	-0.470
Q9D0L7	Armc10	0.027	0.107	-0.131	0.052	0.000	-0.301	-0.431	-0.208
Q9CR61	Ndufb7	0.020	0.088	0.000	-0.043	0.113	0.717	0.435	1.080

Q9DC69	Ndufa9	0.004	0.037	-0.072	0.102	0.000	0.741	0.442	0.635
Q9CQN1	Trap1	0.459	0.719	0.170	0.000	-0.139	0.175	-0.335	-0.254
Q9Z2Z6	Slc25a20	0.006	0.043	0.161	0.000	-0.081	0.953	0.633	0.619
Q921H8	Acaa1a	0.168	0.349	0.077	0.000	-0.377	0.732	-0.098	0.503
Q9ERS2	Ndufa13	0.056	0.168	0.038	-0.179	0.000	0.652	0.143	0.876
P19783	Cox4i1	0.004	0.038	0.100	-0.024	0.000	0.711	0.422	0.696
Q9CZ42	Naxd	0.001	0.018	0.148	0.000	-0.050	1.030	0.765	0.960
Q99LC3	Ndufa10	0.006	0.047	0.000	0.010	-0.249	0.718	0.460	0.860
Q8R2Y8	Ptrh2	0.015	0.077	-0.265	0.190	0.000	1.240	1.150	2.290
Q8R4N0	Clybl	0.061	0.177	0.182	0.000	-0.138	0.704	0.176	0.694
O88696	Clpp	0.016	0.078	0.000	0.043	-0.364	0.863	0.401	0.665
Q80Y81	Elac2	0.624	0.899	0.000	-0.208	0.155	0.131	-0.831	0.116
Q9CRD0	Ociad1	0.184	0.372	-0.379	0.247	0.000	-0.636	-0.622	-0.097
Q64105	Spr	0.003	0.033	-0.370	0.096	0.000	-1.650	-1.710	-1.160
P09528	Fth1	0.085	0.220	0.442	-0.233	0.000	-0.225	-1.190	-0.715
Q9DB77	Uqcrc2	0.140	0.306	0.000	0.125	-0.097	0.556	0.046	0.312
Q61425	Hadh	0.375	0.623	-0.733	0.214	0.000	0.578	0.284	-0.255
Q99MN9	Pccb	0.003	0.032	0.000	0.238	-0.023	0.894	0.712	1.040
Q8BMS1	Hadha	0.932	1.000	0.027	0.000	-0.269	0.492	-0.475	-0.178
P38060	Hmgcl	0.061	0.177	0.000	0.148	-0.178	0.592	0.202	0.328
Q9CZ13	Uqcrc1	0.015	0.076	0.081	0.000	-0.051	0.660	0.292	0.464
Q922W5	Pycr1	0.000	0.014	0.208	0.000	-0.219	2.510	1.920	2.420
P97287	Mcl1	0.225	0.427	0.197	0.000	-0.777	0.487	-0.153	1.300
P50544	Acadvl	0.245	0.456	0.000	0.061	-0.285	0.866	-0.022	0.132
Q8CAQ8	Immt	0.003	0.032	0.000	0.109	-0.145	0.915	0.583	0.795
P56480	Atp5b	0.009	0.057	0.000	0.258	-0.162	0.723	0.568	0.808
Q9CYG7	Tomm34	0.013	0.071	-0.013	0.210	0.000	-0.338	-0.800	-0.588
O35857	Timm44	0.016	0.078	0.231	0.000	-0.162	0.827	0.450	0.765
Q8CC88	Vwa8	0.942	1.000	0.000	0.045	-0.313	0.847	-0.752	-0.481
Q91VD9	Ndufs1	0.002	0.025	0.000	0.197	-0.065	0.847	0.648	0.786
Q8BFR5	Tufm	0.440	0.697	0.165	0.000	-0.339	0.256	-0.698	-0.598
Q14C51	Ptcd3	0.001	0.019	0.000	0.011	-0.187	0.717	0.554	0.741
Q9QX60	Dguok	0.708	0.984	-0.077	0.150	0.000	0.044	-0.050	0.196
Q9JJL8	Sars2	0.446	0.703	0.281	0.000	-0.050	0.559	-0.022	0.196
Q99JR1	Sfxn1	0.018	0.083	0.000	0.180	-0.179	0.403	0.408	0.393
Q80Y14	Glrx5	0.112	0.265	0.079	0.000	-0.218	0.910	0.185	0.266
P50136	Bckdha	0.099	0.243	0.030	0.000	-0.341	0.421	0.014	0.389
P54071	Idh2	0.004	0.036	0.054	0.000	-0.276	1.440	0.889	0.986
Q9WTP7	Ak3	0.037	0.129	-0.146	0.101	0.000	0.809	0.233	0.598
Q6NVE9	Pptc7	0.557	0.825	0.000	-0.054	0.016	0.205	-0.490	-0.140
Q7TMF3	Ndufa12	0.011	0.064	-0.161	0.000	0.141	0.792	0.547	1.110
O35465	Fkbp8	0.521	0.786	0.000	0.167	-0.029	0.317	-0.227	-0.452
Q60932	Vdac1	0.006	0.046	0.000	0.313	-0.052	1.240	1.010	1.700
Q60597	Ogdh	0.007	0.048	0.000	0.044	-0.239	0.747	0.432	0.587
Q78IK4	Apool	0.010	0.060	-0.061	0.205	0.000	0.712	0.459	0.823

Q91YT0	Ndufv1	0.001	0.019	0.000	0.034	-0.076	0.817	0.553	0.688
Q91ZA3	Pcca	0.004	0.037	0.000	0.273	-0.086	0.822	0.667	0.847
Q3UV70	Pdp1	0.009	0.056	-0.168	0.000	0.071	0.763	0.401	0.775
P51174	Acadl	0.039	0.132	0.000	0.343	-0.012	0.526	0.424	0.475
Q8K4Z3	Naxe	0.013	0.071	0.012	0.000	-0.125	0.324	0.340	0.156
Q99KI0	Aco2	0.135	0.299	0.000	0.042	-0.166	0.553	0.087	0.138
P26443	Glud1	0.007	0.048	-0.208	0.207	0.000	0.756	0.579	0.813
Q8BMF4	Dlat	0.002	0.025	0.000	0.245	-0.070	1.060	0.823	1.080
Q9Z2I9	Sucla2	0.144	0.311	0.045	0.000	-0.226	0.968	0.063	0.320
Q9DCS9	Ndufb10	0.006	0.043	0.000	0.250	-0.106	0.628	0.918	0.805
Q99MN1	Kars	0.095	0.237	0.000	0.246	-0.487	-0.474	-0.558	-0.660
Q8R123	Flad1	0.186	0.376	0.107	0.000	-0.001	-0.107	-0.914	-0.144
Q9JMH6	Txnrd1	0.030	0.114	0.000	-0.115	0.093	-0.408	-1.270	-1.320
Q9D2G2	Dlst	0.003	0.033	0.000	0.150	-0.204	0.862	0.626	0.867
Q80ZK0	Mrps10	0.027	0.107	0.000	0.562	-0.484	1.350	0.904	1.690
Q9CRB9	Chchd3	0.005	0.039	-0.052	0.233	0.000	0.868	0.646	1.000
Q9DAM5	Slc25a19	0.512	0.776	0.000	0.235	-0.215	0.146	0.162	-1.530
Q99J39	Mlycd	0.122	0.280	0.000	0.662	-0.127	0.733	0.678	0.583
P08228	Sod1	0.000	0.013	-0.224	0.010	0.000	-1.080	-1.290	-1.170
Q99LC5	Etfa	0.310	0.542	0.000	0.213	-0.198	0.705	-0.164	0.467
Q8BH04	Pck2	0.009	0.056	0.068	0.000	-0.323	1.320	0.741	0.823
Q9Z0V7	Timm17b	0.705	0.982	0.000	-0.059	0.096	0.339	-0.298	0.243
Q9WTQ8	Timm23	0.001	0.017	0.000	0.198	-0.057	0.799	0.774	0.865
P28352	Apex1	0.028	0.107	0.000	-0.288	0.047	-0.452	-0.881	-0.582
Q80XN0	Bdh1	0.208	0.404	0.000	0.147	-0.057	0.047	-0.615	-0.246
Q8BVI4	Qdpr	0.149	0.319	0.000	-0.293	0.016	-0.220	-0.562	-0.276
Q9CQ62	Decr1	0.431	0.687	-0.053	0.110	0.000	0.728	-0.038	0.024
Q9WV85	Nme3	0.093	0.234	0.001	-0.129	0.000	-0.343	-0.578	-0.122
Q99J99	Mpst	0.334	0.571	0.163	0.000	-0.160	0.378	-0.932	-0.891
Q3UQ84	Tars2	0.008	0.053	0.000	0.010	-0.095	0.543	0.329	0.293
Q8CD10	Micu2	0.000	0.014	-0.023	0.080	0.000	1.030	0.770	0.952
Q8JZN5	Acad9	0.215	0.412	0.000	0.153	-0.210	0.571	0.123	0.083
Q91V61	Sfxn3	0.006	0.043	-0.166	0.192	0.000	0.721	0.546	0.762
Q91WD5	Ndufs2	0.006	0.045	0.000	0.406	-0.118	0.946	0.914	1.040
Q8BKZ9	Pdhx	0.019	0.086	0.000	0.072	-0.571	0.880	0.548	0.654
Q8VCF0	Mavs	0.900	1.000	-0.276	0.000	0.068	-0.034	-0.283	0.051
Q60930	Vdac2	0.002	0.023	0.000	0.161	-0.063	1.040	0.946	1.360
Q8K009	Aldh1l2	0.000	0.014	0.838	0.000	-0.233	4.520	3.900	4.030
Q3UJU9	Rmdn3	0.332	0.570	0.215	0.000	-0.020	-0.077	-0.868	0.115
Q61941	Nnt	0.000	0.013	1.030	-0.638	0.000	5.910	5.440	5.700
Q9D0S9	Hint2	0.816	1.000	0.000	1.130	-1.100	-0.268	1.220	-0.316
Q921S7	Mrpl37	0.249	0.462	0.174	0.000	-0.144	0.783	-0.030	0.302
Q3V3R1	Mthfd1l	0.570	0.839	0.201	0.000	-0.215	0.497	-0.751	-0.501
Q8K411	Pitrm1	0.003	0.030	0.219	0.000	-0.224	1.320	0.923	1.190
Q9D1L0	Chchd2	0.596	0.869	-0.345	0.000	0.075	0.112	-1.140	0.029

Q9QYR9	Acot2	0.001	0.018	-0.148	0.041	0.000	0.854	0.676	0.934
Q9JHW2	Nit2	0.309	0.542	-0.141	0.431	0.000	0.296	0.245	0.362
Q9DB20	Atp5o	0.001	0.019	0.000	0.129	-0.067	0.904	0.673	0.901
O08709	Prdx6	0.003	0.031	0.085	0.000	-0.156	-1.090	-1.810	-1.670
Q8BIJ6	Iars2	0.008	0.053	0.000	0.073	-0.104	0.819	0.408	0.655
Q9DC61	Pmpca	0.012	0.068	0.000	0.351	-0.158	1.180	0.802	0.788
Q99L13	Hibadh	0.000	0.014	0.000	0.215	-0.163	1.320	1.240	1.450
Q03265	Atp5a1	0.002	0.025	0.000	0.050	-0.113	0.804	0.540	0.809
Q9QXX4	Slc25a13	0.004	0.038	-0.014	0.047	0.000	0.518	0.393	0.699
O35855	Bcat2	0.331	0.569	0.049	0.000	-0.484	0.516	-0.134	0.039
Q9CQC7	Ndufb4	0.005	0.039	0.000	0.250	-0.034	0.719	0.760	0.569
Q8CGK3	Lonp1	0.159	0.335	0.242	0.000	-0.158	1.280	0.204	0.415
Q8VEM8	Slc25a3	0.001	0.019	0.000	0.026	-0.130	1.170	0.803	0.950
Q9D051	Pdhb	0.332	0.570	0.000	0.004	-0.255	0.792	-0.134	0.064
Q9CYR0	Ssbp1	0.351	0.592	0.000	0.077	0.000	0.385	-0.069	0.185
Q64521	Gpd2	0.010	0.061	0.000	0.125	-0.395	0.903	0.600	0.693
Q9Z0X1	Aifm1	0.917	1.000	0.000	0.036	-0.025	0.160	-0.134	0.015
Q8K1R3	Pnpt1	0.333	0.571	0.128	0.000	-0.456	1.050	0.609	-0.428
Q91V92	Acly	0.836	1.000	0.521	0.000	-0.217	0.528	-0.126	0.096
Q9WUM5	Suclg1	0.579	0.850	0.279	0.000	-0.064	1.160	-0.031	-0.140
Q8K2B3	Sdha	0.017	0.081	0.000	0.002	-0.204	0.722	0.297	0.454
P67778	Phb	0.003	0.033	0.000	0.017	-0.309	0.843	0.579	0.735
Q922Q4	Pycr2	0.016	0.078	0.102	0.000	-0.106	1.190	0.520	0.764
Q61102	Abcb7	0.585	0.857	-0.410	0.169	0.000	0.145	-0.268	0.316
P47802	Mtx1	0.295	0.522	0.000	0.014	-0.282	0.283	-0.076	0.042
P08249	Mdh2	0.023	0.096	-0.067	0.250	0.000	0.700	0.373	0.737
P42125	Eci1	0.192	0.384	0.000	0.429	-0.075	0.361	0.416	0.326
O35143	Atpif1	0.013	0.069	0.259	0.000	-0.321	1.590	1.150	0.813
P84084	Arf5	0.092	0.233	0.000	0.100	-0.067	-0.116	-1.250	-0.806
O35972	Mrpl23	0.003	0.033	0.000	0.107	-0.240	0.886	0.665	0.669
Q9D0D3	Mtpap	0.039	0.134	-0.042	0.000	0.411	0.553	0.544	0.831
Q9D6S7	Mrrf	0.086	0.223	0.000	0.433	-0.651	0.785	0.917	0.434
Q9CXT8	Pmpcb	0.017	0.082	0.000	0.156	-0.441	1.050	0.643	0.643
Q8CHT0	Aldh4a1	0.875	1.000	0.335	-0.067	0.000	1.090	-0.715	-0.397
Q9D1N9	Mrpl21	0.001	0.019	0.000	0.089	-0.218	0.892	0.811	1.010
Q8QZT1	Acat1	0.050	0.156	0.000	0.181	-0.006	0.765	0.228	0.592
Q9DCS3	Mecr	0.121	0.279	0.000	0.207	-0.206	1.210	0.269	0.392
Q3ULD5	Mccc2	0.004	0.036	0.000	0.315	-0.197	1.080	0.893	0.981
Q9DCC8	Tomm20	0.641	0.917	-0.090	0.350	0.000	0.068	0.058	0.398
P55096	Abcd3	0.092	0.232	0.042	0.000	-0.929	1.360	1.060	0.053
P32020	Scp2	0.103	0.248	0.172	0.000	-0.126	0.611	0.091	0.465
P19096	Fasn	0.558	0.826	0.157	0.000	-0.066	0.282	-0.464	-0.161
Q8BH95	Echs1	0.035	0.124	0.000	0.240	-0.097	0.915	0.414	0.524
P56391	Cox6b1	0.015	0.076	0.029	0.000	-0.083	0.406	0.174	0.409
Q99N96	Mrpl1	0.091	0.231	0.000	-1.980	0.201	0.820	0.852	1.270

Q99KE1	Me2	0.952	1.000	0.000	0.140	-0.205	0.190	-0.212	-0.072
Q60931	Vdac3	0.007	0.050	0.000	0.252	-0.058	1.080	0.902	1.570
P09925	Surf1	0.000	0.013	0.069	0.000	-0.073	0.682	0.590	0.722
Q8BMD8	Slc25a24	0.023	0.096	-0.447	0.176	0.000	0.526	0.574	0.757
P11352	Gpx1	0.131	0.294	0.457	0.000	-0.235	-0.028	-0.804	-0.877
P54116	Stom	0.556	0.825	0.677	0.000	-0.243	0.446	-0.539	-0.245
P99029	Prdx5	0.996	1.000	0.234	0.000	-0.266	0.051	-0.201	0.121
Q07417	Acads	0.730	1.000	0.089	0.000	-0.125	0.435	-0.537	-0.263
P12787	Cox5a	0.002	0.028	0.000	0.139	-0.028	0.629	0.454	0.629
Q9D0M3	Cyc1	0.192	0.384	0.243	0.000	-0.038	0.016	0.587	0.597
Q80X85	Mrps7	0.051	0.158	0.000	0.066	-0.627	0.765	0.358	0.418
P35486	Pdha1	0.264	0.482	0.044	0.000	-0.235	0.792	0.073	-0.010
Q1HFZ0	Nsun2	0.102	0.247	0.289	0.000	-0.086	-0.029	-1.110	-0.849
Q61171	Prdx2	0.040	0.134	0.088	0.000	-0.208	-0.353	-0.755	-0.402
Q8K3A0	Hscb	0.378	0.627	0.020	0.000	-7.410	-0.071	0.082	-0.054
Q9D6K5	Synj2bp	0.056	0.167	0.000	0.262	-0.061	0.573	0.279	0.805
Q61576	Fkbp10	0.000	0.014	0.444	-0.772	0.000	4.040	3.680	3.950
Q9QZD8	Slc25a10	0.003	0.030	0.000	0.214	-0.415	1.190	1.260	1.510
Q61733	Mrps31	0.132	0.296	0.000	0.114	-0.108	0.841	-0.005	0.687
Q9CQH3	Ndufb5	0.003	0.032	0.000	0.202	-0.151	0.682	0.684	0.812
Q9Z1J3	Nfs1	0.866	1.000	0.239	0.000	-0.042	0.878	-0.349	-0.124
P58059	Mrps21	0.054	0.163	0.000	0.089	-0.066	0.647	0.140	0.589
P53395	Dbt	0.046	0.149	0.000	0.286	-0.049	0.968	0.326	0.859
Q9ESW4	Agk	0.183	0.372	0.000	-0.572	0.077	0.527	-0.141	0.703
O89110	Casp8	0.012	0.067	0.166	-0.032	0.000	-1.050	-1.120	-2.060
Q9CZ83	Mrpl55	0.003	0.031	0.173	0.000	-0.267	0.903	0.758	0.928
Q9DCT2	Ndufs3	0.021	0.090	-0.343	0.080	0.000	0.597	0.506	1.070
P51660	Hsd17b4	0.034	0.122	0.027	0.000	-0.287	1.110	0.293	0.795
Q8VCW8	Acsf2	0.374	0.623	0.347	-0.053	0.000	0.597	-1.130	-0.804
Q8K1J6	Trnt1	0.089	0.227	-0.056	0.000	0.076	-0.250	-0.666	-0.153
Q9CPR5	Mrpl15	0.032	0.119	0.144	-1.190	0.000	1.030	0.900	1.190
Q9DCW4	Etfb	0.322	0.558	0.252	0.000	-0.128	0.867	-0.052	0.291
Q8VD26	Tmem143	0.001	0.022	-0.252	0.062	0.000	0.775	0.891	1.040
Q9CQ54	Ndufc2	0.001	0.022	0.044	0.000	-0.180	0.889	0.645	0.923
Q9CQ75	Ndufa2	0.016	0.078	-0.063	0.189	0.000	0.742	0.367	0.640
P09671	Sod2	0.023	0.096	-0.061	0.348	0.000	0.715	0.476	0.682
P08074	Cbr2	0.001	0.019	0.000	0.042	-1.580	5.280	4.340	4.610
Q99LP6	Grpel1	0.003	0.030	0.000	0.185	-0.177	1.010	0.736	0.977
Q9CR62	Slc25a11	0.026	0.105	0.013	0.000	-0.081	0.658	0.233	0.369
P45952	Acadm	0.441	0.697	0.020	0.000	-0.423	0.852	-0.186	-0.129
Q80ZS3	Mrps26	0.050	0.155	-0.354	0.562	0.000	0.950	0.666	0.952
Q9Z2Q5	Mrpl40	0.003	0.031	0.154	-0.056	0.000	0.713	0.525	0.546
Q924T2	Mrps2	0.014	0.073	0.000	0.348	-0.330	0.736	1.060	0.937
Q9CQA3	Sdhb	0.005	0.042	0.000	0.126	-0.143	0.648	0.433	0.552
Q9D3P8	Plgrkt	0.012	0.069	-0.004	0.232	0.000	0.479	0.379	0.528

P05202	Got2	0.002	0.028	0.000	0.188	-0.073	0.799	0.617	0.665
O08756	Hsd17b10	0.087	0.224	-0.012	0.447	0.000	0.862	0.393	0.563
Q64133	Maoa	0.000	0.011	0.151	0.000	-0.761	3.640	3.580	3.770
P22315	Fech	0.179	0.364	0.378	-1.300	0.000	1.010	0.491	0.277
Q9CPQ8	Atp5l	0.001	0.022	-0.234	0.085	0.000	0.683	0.763	0.798
Q3UMR5	Mcu	0.004	0.038	0.000	0.207	-0.231	0.923	0.701	0.918
Q9WUR2	Eci2	0.392	0.641	-0.002	0.400	0.000	0.637	0.088	0.274
Q61578	Fdxr	0.099	0.243	0.000	0.172	-0.061	-0.043	-0.801	-0.540
Q8JZQ2	Afg3l2	0.015	0.076	0.000	0.089	-0.109	0.828	0.358	0.590
Q9D880	Timm50	0.034	0.123	0.000	0.165	-0.056	0.558	0.221	0.486
Q9D855	Uqcrb	0.013	0.069	0.000	0.173	-0.233	0.669	0.448	0.554
Q5M8N4	Sdr39u1	0.975	1.000	-0.090	1.850	0.000	-0.753	1.040	1.390
P19536	Cox5b	0.287	0.512	-0.201	0.104	0.000	0.204	-0.054	0.216
Q791V5	Mtch2	0.007	0.048	0.000	0.565	-0.017	1.160	1.140	1.190
Q8BFP9	Pdk1	0.386	0.634	0.043	0.000	-0.101	0.101	-0.625	-0.165
Q9EQ20	Aldh6a1	0.368	0.615	0.300	0.000	-0.150	1.460	-0.061	0.226
Q8K2M0	Mrpl38	0.002	0.024	0.114	0.000	-0.089	0.995	0.671	0.855
P16125	Ldhb	0.000	0.013	-0.212	0.132	0.000	-1.330	-1.570	-1.550
Q9DC50	Crot	0.119	0.276	0.000	-0.124	0.034	-0.050	-0.367	-0.340
O35435	Dhodh	0.072	0.196	0.000	0.359	-1.330	0.638	1.240	1.160
O88441	Mtx2	0.276	0.496	0.000	0.113	-0.108	0.703	-0.135	0.393
Q8BJ03	Cox15	0.015	0.075	0.000	-0.638	0.346	1.240	1.100	1.810
Q8R164	Bphl	0.031	0.114	0.197	0.000	-0.133	0.714	0.305	0.778
Q920E5	Fdps	0.104	0.252	1.020	0.000	-0.336	1.880	0.878	1.120
Q9DBF1	Aldh7a1	0.000	0.009	0.000	0.318	-0.191	2.700	2.460	2.520
Q99MR8	Mccc1	0.382	0.631	0.014	0.000	-0.317	0.811	-0.251	0.108
Q9WVL0	Gstz1	0.112	0.265	0.000	0.163	-0.024	1.190	0.487	0.239
Q922H2	Pdk3	0.193	0.385	0.065	0.000	-0.219	0.781	-0.001	0.229
Q9JMA2	Qtrt1	0.001	0.019	0.000	0.059	-0.158	-0.700	-0.758	-0.891
Q8VCX5	Micu1	0.567	0.836	-0.165	0.177	0.000	1.300	-0.698	0.513
Q99M87	Dnaja3	0.029	0.112	0.003	0.000	-0.329	0.713	0.216	0.625
Q9CQ40	Mrpl49	0.092	0.233	0.339	0.000	-0.837	0.637	0.883	0.445
Q8R3F5	Mcat	0.181	0.368	0.020	0.000	-0.178	0.816	-0.052	0.333
Q9DCN2	Cyb5r3	0.000	0.005	0.000	0.161	-0.051	2.080	1.950	2.120
Q9DB15	Mrpl12	0.006	0.044	0.000	0.110	-0.240	0.803	0.551	0.609
Q99NB1	Acss1	0.101	0.247	0.000	0.064	-0.261	-0.149	-0.573	-0.593
Q9EQI8	Mrpl46	0.017	0.081	0.194	0.000	-0.083	1.000	0.471	0.691
Q8C163	Exog	0.071	0.194	-0.225	0.654	0.000	0.703	0.765	1.070
Q5U458	Dnajc11	0.066	0.185	0.771	-0.042	0.000	1.780	1.580	0.657
O08600	Endog	0.058	0.171	-0.172	0.406	0.000	-0.439	-0.313	-0.402
O35459	Ech1	0.045	0.146	0.000	0.120	-0.262	0.291	0.279	0.264
P47968	Rpia	0.160	0.337	0.718	-0.577	0.000	-0.741	-2.220	-0.389
Q8VDC0	Lars2	0.390	0.640	0.288	0.000	-4.090	0.945	-0.292	-0.213
Q3UHB1	Nt5dc3	0.001	0.014	0.000	0.448	-0.077	2.160	2.160	2.560
Q9DCJ5	Ndufa8	0.075	0.202	0.000	1.300	-0.142	1.220	1.620	2.010

Q9R257	Hebp1	0.892	1.000	0.000	0.167	-1.940	1.520	-2.900	-1.030
Q921G7	Etfdh	0.009	0.055	0.011	0.000	-0.217	0.785	0.394	0.573
Q9JHS4	Clpx	0.557	0.825	0.335	0.000	-0.328	1.540	-0.635	0.363
Q8BK72	Mrps27	0.012	0.068	0.000	0.528	-0.256	1.200	1.050	1.510
Q8BW75	Maob	0.000	0.013	0.667	0.000	-0.370	4.120	3.720	4.010
Q922S4	Pde2a	0.073	0.199	0.280	-0.058	0.000	-0.909	-2.760	-0.790
P48962	Slc25a4	0.000	0.013	0.000	0.359	-0.136	2.120	1.860	2.070
Q9CQX2	Cyb5b	0.013	0.071	-0.164	0.064	0.000	0.975	0.423	0.947
Q9CPQ3	Tomm22	0.010	0.061	-0.029	0.000	0.186	1.040	0.523	0.946
P70349	Hint1	0.003	0.030	-0.064	0.000	0.148	-1.010	-1.670	-1.260
P18155	Mthfd2	0.034	0.122	0.000	0.357	-0.941	0.795	1.290	1.380
Q9DCZ4	Apoo	0.004	0.037	0.000	0.124	-0.197	0.733	0.566	0.860
Q9DCM0	Ethe1	0.636	0.912	0.000	0.137	-0.820	0.135	-0.445	0.180
Q99N93	Mrpl16	0.172	0.355	0.000	0.020	-0.274	0.737	-0.068	0.326
P62897	Cycs	0.205	0.401	0.000	0.081	-0.533	-0.300	-0.469	-0.708
Q9D964	Gatm	0.821	1.000	0.770	0.000	-0.380	0.678	0.053	-0.044
P31786	Dbi	0.001	0.019	0.000	0.100	-0.167	-1.290	-1.720	-1.290
Q9DCU6	Mrpl4	0.041	0.138	0.000	0.039	-0.885	0.969	0.968	0.394
Q9CR58	Slc25a30	0.011	0.062	0.000	0.243	-0.162	0.866	0.561	0.664
Q791T5	Mtch1	0.001	0.019	0.000	0.409	-0.005	1.910	1.480	1.660
Q9DB70	Fundc1	0.256	0.470	-0.517	1.650	0.000	2.470	0.712	1.270
Q9CXZ1	Ndufs4	0.012	0.067	-0.105	0.323	0.000	0.881	0.660	1.120
Q9JK81	Myg1	0.012	0.068	-0.941	0.000	0.003	-1.670	-1.890	-1.610
Q9CQN7	Mrpl41	0.001	0.019	-0.057	0.242	0.000	1.080	0.872	1.000
O35129	Phb2	0.003	0.030	0.000	0.062	-0.164	0.922	0.591	0.718
P97823	Lypla1	0.168	0.350	0.000	-0.250	0.196	0.058	-1.140	-0.961
Q9CXW2	Mrps22	0.536	0.803	0.837	-1.070	0.000	1.960	1.660	-1.400
Q91VT4	Cbr4	0.890	1.000	0.000	0.188	-0.136	0.418	-0.152	-0.122
Q9JKF7	Mrpl39	0.494	0.759	0.000	-3.790	0.010	1.100	-0.144	-1.440
Q91VR2	Atp5c1	0.035	0.125	0.127	0.000	-0.028	1.150	0.535	0.475
Q62425	Ndufa4	0.219	0.419	-0.214	0.210	0.000	0.195	0.073	0.541
Q9D5T0	Atad1	0.387	0.636	0.005	-0.010	0.000	0.504	-0.072	0.068
Q9JKX6	Nudt5	0.000	0.014	0.033	-0.029	0.000	-1.110	-1.520	-1.260
Q8QZS1	Hibch	0.404	0.655	0.000	0.318	-0.092	0.156	0.135	0.318
Q99L04	Dhrs1	0.470	0.729	0.040	0.000	-0.227	0.315	-0.898	-0.478
Q99KB8	Hagh	0.754	1.000	0.142	0.000	-0.442	-0.018	-0.512	-0.014
Q9JLJ2	Aldh9a1	0.081	0.213	0.030	-0.088	0.000	-0.239	-1.410	-0.779
Q91VS7	Mgst1	0.030	0.113	0.999	0.000	-0.245	1.580	1.500	2.410
Q91VM9	Ppa2	0.070	0.193	-0.028	0.474	0.000	0.613	0.457	0.874
Q9D8P4	Mrpl17	0.062	0.179	-0.135	0.609	0.000	0.806	0.896	0.632
P47791	Gsr	0.018	0.084	0.341	0.000	-0.118	-0.466	-1.080	-1.080
Q9JLT4	Txnrd2	0.839	1.000	0.000	0.351	-0.041	0.125	0.029	0.247
Q9CQ69	Uqcrq	0.004	0.038	0.000	0.165	-0.022	0.638	0.470	0.464
Q9CZU6	Cs	0.005	0.041	0.000	0.137	-0.034	0.868	0.509	0.669
O88967	Yme1l1	0.017	0.081	0.000	-0.237	0.021	0.822	0.328	0.838

Q811U4	Mfn1	0.420	0.674	0.000	1.260	-0.739	2.090	1.140	-0.275
Q99J47	Dhrs7b	0.010	0.059	0.000	0.380	-0.485	1.100	1.150	1.120
Q9D6J5	Ndufb8	0.039	0.132	-0.884	0.149	0.000	0.958	0.797	0.597
Q8BJ64	Chdh	0.375	0.623	0.970	-0.135	0.000	0.659	-1.190	-0.567
Q9CQN6	Tmem14c	0.066	0.186	0.146	0.000	-0.210	0.760	0.282	0.302
Q9CQL5	Mrpl18	0.012	0.067	-0.027	0.185	0.000	0.925	0.512	1.060
Q8R2Q4	Gfm2	0.142	0.309	0.000	0.144	-0.128	0.042	-1.100	-0.903
Q8JZU2	Slc25a1	0.000	0.012	0.000	0.212	-0.083	1.600	1.430	1.730
Q8BU88	Mrpl22	0.017	0.080	0.003	-0.068	0.000	0.895	0.346	0.595
P53702	Hccs	0.001	0.022	-0.130	0.313	0.000	1.420	1.460	1.860
P36552	Cpox	0.116	0.271	0.330	0.000	-0.246	-0.140	-0.571	-0.479
Q9D0G0	Mrps30	0.012	0.068	0.000	0.044	-0.091	1.010	0.479	0.609
Q9QYA2	Tomm40	0.010	0.059	0.000	0.202	-0.235	1.300	0.723	0.891
Q8BYM8	Cars2	0.412	0.665	0.076	0.000	-0.350	0.743	-0.258	0.119
Q8BTX9	Hsdl1	0.769	1.000	0.000	0.163	-0.252	0.135	-0.129	0.040
Q8JZN7	Rhot2	0.010	0.061	0.084	0.000	-0.204	0.554	0.327	0.480
Q9CXJ4	Abcb8	0.014	0.074	0.000	0.642	-0.065	1.350	1.020	1.280
Q91WS0	Cisd1	0.396	0.646	-0.215	1.180	0.000	0.469	0.808	0.987
O09174	Amacr	0.030	0.113	0.000	0.612	-0.235	1.240	0.949	0.900
Q9D7N3	Mrps9	0.122	0.280	0.267	-0.306	0.000	1.060	0.047	1.140
Q8BWM0	Ptges2	0.946	1.000	-1.850	2.870	0.000	1.240	0.282	-0.189
Q99PU8	Dhx30	0.416	0.670	0.000	0.380	-0.875	1.330	-0.133	-0.053
Q3URS9	Ccdc51	0.007	0.048	0.194	0.000	-0.221	0.635	0.568	0.670
Q9DC70	Ndufs7	0.005	0.040	0.000	0.110	-0.184	0.684	0.466	0.575
Q9EP89	Lactb	0.009	0.055	0.000	0.227	-0.004	0.611	0.438	0.673
Q9D3D9	Atp5d	0.126	0.287	-0.224	0.095	0.000	0.573	0.056	0.267
O88986	Gcat	0.640	0.917	-0.398	0.000	0.308	0.250	-0.442	-0.349
Q9JHI5	Ivd	0.333	0.571	0.000	0.127	-0.011	0.544	-0.052	0.212
Q9CPP6	Ndufa5	0.245	0.456	-0.536	0.555	0.000	0.694	0.147	0.649
O55125	Nipsnap1	0.039	0.134	-0.109	0.000	0.160	0.335	0.199	0.384
Q8BX10	Pgam5	0.212	0.409	-0.303	0.066	0.000	0.400	-0.081	0.256
P48410	Abcd1	0.804	1.000	0.401	-0.082	0.000	0.518	-0.244	0.258
Q9WTP6	Ak2	0.009	0.057	0.187	0.000	-0.011	-0.394	-0.833	-0.616
Q8BG51	Rhot1	0.015	0.076	0.000	0.146	-0.220	0.857	0.556	0.462
Q60759	Gcdh	0.450	0.708	0.000	0.355	-0.371	0.773	-0.053	0.092
P20108	Prdx3	0.006	0.046	0.214	-0.058	0.000	0.778	0.509	0.675
Q3U2A8	Vars2	0.089	0.227	0.000	0.110	-0.376	0.144	0.368	0.307
Q80YD1	Supv3l1	0.046	0.149	0.000	0.195	-0.039	0.533	0.318	0.254
Q99KK9	Hars2	0.327	0.564	0.000	0.815	-0.658	0.593	0.065	1.500
Q91V12	Acot7	0.020	0.089	-0.038	0.193	0.000	0.736	0.449	0.396
Q8BHF7	Pgs1	0.017	0.080	0.000	1.120	-0.175	2.030	1.790	2.010
Q8K3J1	Ndufs8	0.002	0.025	0.000	0.081	-0.026	0.747	0.504	0.553
Q64433	Hspe1	0.001	0.018	0.000	0.100	-0.080	1.090	0.779	0.991
Q8K0D5	Gfm1	0.253	0.467	0.000	0.253	-0.248	1.020	0.078	0.217
Q99N94	Mrpl9	0.093	0.234	0.142	0.000	-0.854	1.030	0.602	0.221

Q8C3X2	Ccdc90b	0.005	0.040	-0.005	0.303	0.000	1.660	1.020	1.250
Q99N87	Mrps5	0.000	0.012	0.039	0.000	-0.040	1.140	0.934	0.888
P51881	Slc25a5	0.203	0.400	0.000	1.090	-0.121	0.477	1.260	1.440
Q8R1S0	Coq6	0.178	0.364	0.000	0.621	-0.148	0.936	0.515	0.414
Q9DBL7	Coasy	0.134	0.298	7.440	-0.978	0.000	7.090	7.330	7.010
Q5HZI9	Slc25a51	0.018	0.083	-0.031	0.632	0.000	1.200	0.956	1.100
Q91VC9	Ghitm	0.003	0.030	0.000	0.088	-0.147	1.130	0.700	0.953
Q8CAK1	Iba57	0.322	0.558	0.036	0.000	-0.449	0.439	-0.205	0.176
Q9D6K8	Fundc2	0.013	0.071	0.000	0.474	-0.255	1.690	1.410	0.958
O08807	Prdx4	0.256	0.470	0.038	0.000	-0.002	0.439	-0.070	0.262
Q9QZ23	Nfu1	0.022	0.094	0.000	0.235	-0.727	1.080	0.755	1.350
P51175	Ppox	0.909	1.000	0.000	0.082	-0.711	0.651	-0.730	-0.373
Q9JHR7	Ide	0.004	0.036	0.000	0.341	-0.020	-0.764	-0.819	-0.576
Q9D1P0	Mrpl13	0.522	0.787	0.000	0.348	-0.702	0.959	-0.679	0.596
Q8K1M6	Dnm1l	0.892	1.000	0.042	0.000	-0.313	0.147	-0.185	-0.310
Q8K4X7	Agpat4	0.005	0.042	0.000	0.300	-0.338	1.030	0.953	1.130
Q9CZL5	Pcbd2	0.058	0.171	0.028	0.000	-0.711	0.373	0.407	0.453
P48771	Cox7a2	0.957	1.000	-0.456	0.000	0.309	0.089	-0.409	0.125
P70404	Idh3g	0.775	1.000	0.000	0.070	-0.248	0.756	-0.504	-0.078
Q9D6Y7	Msra	0.068	0.189	0.549	-0.217	0.000	-0.252	-0.768	-0.814
P40630	Tfam	0.889	1.000	0.483	0.000	-0.272	0.049	0.003	0.264
P97386	Lig3	0.578	0.849	0.068	0.000	-0.173	0.026	-0.596	0.058
Q4VAE3	Tmem65	0.014	0.072	0.000	0.487	-0.336	1.190	1.030	1.570
P56135	Atp5j2	0.005	0.040	0.000	0.393	-0.087	0.924	1.130	0.968
Q9DCM2	Gstk1	0.701	0.977	0.010	0.000	-0.212	0.377	-0.571	-0.378
Q9D0C4	Trmt5	0.109	0.260	-0.090	0.320	0.000	-0.850	-4.670	-1.540
Q924L1	Letmd1	0.672	0.950	-1.210	0.847	0.000	-1.630	0.966	-1.040
Q9D8S4	Rexo2	0.107	0.257	-0.191	0.000	0.052	0.356	0.007	0.431
Q7TSQ8	Pdpr	0.166	0.347	0.000	0.234	-0.035	1.070	0.066	0.599
Q9D7B6	Acad8	0.170	0.353	0.000	-1.970	0.001	1.380	0.114	0.357
Q8K370	Acad10	0.053	0.162	0.324	0.000	-0.339	0.796	0.651	0.382
P70677	Casp3	0.003	0.030	-0.016	0.138	0.000	-1.190	-2.040	-1.570
P61922	Abat	0.014	0.073	0.000	1.570	-1.050	4.160	3.100	3.540
Q9CQZ6	Ndufb3	0.002	0.025	-0.037	0.054	0.000	0.800	0.535	0.580
Q9D6M3	Slc25a22	0.001	0.016	0.000	0.088	-0.280	1.510	1.170	1.410
Q9D2R6	Coa3	0.017	0.080	0.000	0.304	-0.248	1.120	0.611	0.904
P47934	Crat	0.395	0.644	0.000	-3.600	0.145	2.180	-1.640	0.743
Q91WK1	Spryd4	0.045	0.146	0.000	0.231	-0.354	0.951	0.398	0.566
Q9CQE1	Nipsnap3b	0.179	0.365	0.000	0.874	-0.677	0.769	0.963	0.692
Q9WVD5	Slc25a15	0.005	0.042	-0.508	0.364	0.000	1.270	1.750	1.660
Q91Z53	Grhpr	0.060	0.176	0.000	-0.087	0.077	-0.125	-0.652	-0.656
Q91VA6	Poldip2	0.121	0.278	0.000	0.211	-1.280	1.200	0.544	0.335
Q9Z1P6	Ndufa7	0.004	0.037	-0.044	0.000	0.010	1.000	0.554	0.732
P58281	Opa1	0.111	0.263	-0.010	0.051	0.000	-0.089	-0.563	-0.194
Q8VDT9	Mrpl50	0.179	0.365	0.195	-2.670	0.000	0.890	0.464	0.716

Q8K215	Lyrm4	0.019	0.086	0.000	0.224	-0.231	0.718	0.439	0.602
Q9JLZ3	Auh	0.243	0.453	0.028	0.000	-0.092	0.540	-0.170	0.570
Q8BGA9	Oxa1l	0.039	0.133	0.000	-1.550	0.191	1.400	1.020	1.330
Q8BUY5	Timmdc1	0.012	0.067	0.000	0.616	-0.099	1.080	1.290	1.470
Q9ERB0	Snap29	0.440	0.697	0.870	-3.570	0.000	0.006	0.284	0.520
O54918	Bcl2l11	0.580	0.851	0.000	0.199	-0.024	0.457	-0.129	0.176
Q9D8Y1	Tmem126a	0.000	0.011	0.000	0.009	-0.058	1.380	1.100	1.350
Q3TL44	Nlrx1	0.072	0.196	0.000	0.222	-0.158	0.479	0.242	0.303
Q9CZN8	Qrsl1	0.705	0.982	0.000	0.121	-0.527	0.510	-0.341	-0.174
Q91VN4	Chchd6	0.012	0.068	0.000	0.253	-1.110	1.520	1.500	1.590
Q9WU56	Pus1	0.903	1.000	2.900	-0.702	0.000	2.620	0.251	-1.290
Q71RI9	Kyat3	0.016	0.079	0.000	0.041	-0.711	0.821	0.680	0.805
O88587	Comt	0.014	0.072	-0.457	0.392	0.000	1.030	0.947	1.110
Q9D338	Mrpl19	0.721	1.000	0.000	-0.168	0.031	1.100	-0.582	-0.080
Q8BIP0	Dars2	0.009	0.056	0.571	-0.292	0.000	1.550	1.440	2.180
Q99LB2	Dhrs4	0.046	0.147	0.000	0.173	-0.424	1.020	0.369	0.601
Q8C5H8	Nadk2	0.914	1.000	0.228	0.000	-0.004	0.494	-0.258	-0.095
Q9CQE3	Mrps17	0.067	0.187	0.000	1.020	-0.199	0.968	1.420	1.540
Q9JKL4	Ndufaf3	0.124	0.284	0.000	-1.040	0.623	0.737	0.694	1.040
Q8BHC4	Dcakd	0.017	0.081	0.466	0.000	-1.560	2.210	1.810	2.370
Q9D1I6	Mrpl14	0.431	0.687	0.279	0.000	-0.206	0.925	-0.016	0.044
P41216	Acsl1	0.926	1.000	0.000	0.371	-0.869	0.044	0.090	-0.768
P52503	Ndufs6	0.095	0.237	-0.325	0.000	0.739	0.967	0.626	1.040
Q3U186	Rars2	0.906	1.000	0.398	-0.283	0.000	1.060	-0.833	-0.334
Q3U5Q7	Cmpk2	0.171	0.354	0.545	0.000	-0.063	0.143	-0.649	-0.640
Q8BK08	Tmem11	0.001	0.019	-0.043	0.285	0.000	1.080	0.954	1.080
Q60649	Clpb	0.990	1.000	0.421	0.000	-0.108	0.340	-0.304	0.289
Q3TC33	Ccdc127	0.031	0.114	-0.180	0.197	0.000	0.651	0.308	0.506
Q9CY73	Mrpl44	0.009	0.057	0.000	0.358	-0.175	0.827	0.770	0.815
Q80U63	Mfn2	1.000	1.000	0.000	0.057	-0.064	-0.674	0.298	0.369
Q9DC71	Mrps15	0.711	0.987	-0.194	0.000	0.044	0.841	-0.657	0.193
Q9CR59	Gadd45gip1	0.130	0.293	0.000	0.151	-0.194	0.895	0.031	0.578
Q8BVU5	Nudt9	0.155	0.330	0.000	-0.351	0.224	0.896	2.140	0.088
Q2TPA8	Hsdl2	0.861	1.000	0.190	-0.464	0.000	0.705	-0.463	-0.284
Q9D8S9	Bola1	0.460	0.719	0.000	-2.370	0.187	1.050	-0.214	-0.646
Q9CR68	Uqcrfs1	0.007	0.048	0.000	0.074	-0.106	0.656	0.355	0.524
Q66GT5	Ptpmt1	0.058	0.172	0.000	0.711	-0.229	1.030	0.782	0.979
Q9CQZ5	Ndufa6	0.233	0.438	0.000	0.678	-0.132	0.410	0.479	0.879
Q99N89	Mrpl43	0.003	0.030	0.000	0.185	-0.319	1.180	0.974	1.330
P56382	Atp5e	0.000	0.013	0.000	0.115	-0.081	0.914	0.754	0.858
Q9WV84	Nme4	0.027	0.105	1.300	-1.640	0.000	3.340	2.630	2.720
Q9D1B9	Mrpl28	0.304	0.534	0.000	0.671	-1.410	0.836	0.095	0.635
Q8BIG7	Comtd1	0.937	1.000	0.000	0.166	-0.276	0.673	-0.609	-0.276
Q5IRJ6	Slc30a9	0.003	0.030	0.000	0.577	-0.728	2.360	2.540	2.810
Q924D0	Rtn4ip1	0.540	0.807	0.000	0.155	-0.927	0.162	-2.980	-0.090

Q9D023	Mpc2	0.034	0.121	0.000	0.091	-0.908	0.954	0.558	0.976
Q8K2Y7	Mrpl47	0.048	0.152	0.000	0.273	-0.157	1.070	0.895	0.325
Q9CPQ1	Cox6c	0.008	0.052	0.000	0.148	-0.102	0.358	0.542	0.504
Q99N85	Mrps18a	0.010	0.060	0.176	-0.091	0.000	0.951	0.506	0.708
P63030	Mpc1	0.002	0.028	0.158	-0.024	0.000	1.540	0.979	1.210
Q921N7	Tmem70	0.029	0.111	-0.295	3.890	0.000	5.300	5.780	6.340
Q9CYK1	Wars2	0.461	0.720	-0.361	0.518	0.000	0.617	-0.291	0.913
Q61586	Gpam	0.000	0.012	0.000	-0.259	0.263	2.000	1.940	2.100
Q8BYL4	Yars2	0.539	0.807	0.319	-1.240	0.000	0.125	-1.890	-0.671
Q9ERI6	Rdh14	0.189	0.379	0.000	0.132	-0.126	-0.072	-0.191	-0.680
Q9D6U8	Fam162a	0.000	0.013	-0.044	0.195	0.000	1.030	0.901	0.963
Q9D125	Mrps25	0.125	0.285	5.570	-3.410	0.000	6.160	5.650	5.610
Q9DC29	Abcb6	0.117	0.272	0.022	-0.201	0.000	-0.174	-0.821	-0.399
Q9CZ57	Nsun4	0.430	0.686	0.129	-1.190	0.000	0.559	-0.895	1.310
Q9DAT5	Trmu	0.965	1.000	0.330	-0.121	0.000	0.746	-0.391	-0.095
Q9D1R1	Tmem126b	0.441	0.698	0.146	-0.371	0.000	-0.573	0.779	0.798
Q3TQB2	Foxred1	0.796	1.000	-0.086	0.930	0.000	0.212	0.095	0.873
Q9CQ91	Ndufa3	0.595	0.868	-0.286	0.062	0.000	0.320	-0.326	0.166
Q99N95	Mrpl3	0.067	0.187	0.000	0.558	-0.395	1.000	0.576	0.857
Q9DB10	Smdt1	0.087	0.224	-0.402	0.296	0.000	0.541	0.458	0.326

Supp. Table 2. Mitochondrial proteome was detected in CD4$^+$ T cells isolated from old mice after mito-transfer and in non-manipulated CD4$^+$ T cells from old mice. CD4$^+$ T cells from young and old mice, and from old mice after mito-transfer were cultured for 4 h before processing for mass spectrometry analysis. Data expressed as median protein Log2 fold change of CD4$^+$ T cells from old mice, from 3 individual old mice (paired experiment)

Uniprot.ID	Gene.ID	P. Val	FDR	O1	O2	O3	OM1	OM2	OM3
Q8JZN5	Acad9	0.215	0.412	0.000	0.150	-0.210	0.570	0.120	0.080
Q9Z0X1	Aifm1	0.917	1.000	0.000	0.040	-0.030	0.160	-0.130	0.010
Q3TQB2	Foxred1	0.796	1.000	-0.090	0.930	0.000	0.210	0.100	0.870
Q99LC3	Ndufa10	0.006	0.047	0.000	0.010	-0.250	0.720	0.460	0.860
Q7TMF3	Ndufa12	0.011	0.064	-0.160	0.000	0.140	0.790	0.550	1.110
Q9ERS2	Ndufa13	0.056	0.168	0.040	-0.180	0.000	0.650	0.140	0.880
Q9CQ75	Ndufa2	0.016	0.078	-0.060	0.190	0.000	0.740	0.370	0.640

Q9CQ91	Ndufa3	0.595	0.868	-0.290	0.060	0.000	0.320	-0.330	0.170
Q9CPP6	Ndufa5	0.245	0.456	-0.540	0.560	0.000	0.690	0.150	0.650
Q9CQZ5	Ndufa6	0.233	0.438	0.000	0.680	-0.130	0.410	0.480	0.880
Q9Z1P6	Ndufa7	0.004	0.037	-0.040	0.000	0.010	1.000	0.550	0.730
Q9DCJ5	Ndufa8	0.075	0.202	0.000	1.300	-0.140	1.220	1.620	2.010
Q9DC69	Ndufa9	0.004	0.037	-0.070	0.100	0.000	0.740	0.440	0.640
Q9JKL4	Ndufaf3	0.124	0.284	0.000	-1.040	0.620	0.740	0.690	1.040
Q9DCS9	Ndufb10	0.006	0.043	0.000	0.250	-0.110	0.630	0.920	0.810
O09111	Ndufb11	0.001	0.018	0.020	-0.200	0.000	0.580	0.570	0.650
Q9CQZ6	Ndufb3	0.002	0.025	-0.040	0.050	0.000	0.800	0.540	0.580
Q9CQC7	Ndufb4	0.005	0.039	0.000	0.250	-0.030	0.720	0.760	0.570
Q9CQH3	Ndufb5	0.003	0.032	0.000	0.200	-0.150	0.680	0.680	0.810
Q9CR61	Ndufb7	0.020	0.088	0.000	-0.040	0.110	0.720	0.440	1.080
Q9D6J5	Ndufb8	0.039	0.132	-0.880	0.150	0.000	0.960	0.800	0.600
Q9CQJ8	Ndufb9	0.003	0.033	-0.200	0.220	0.000	0.740	0.740	0.810
Q9CQ54	Ndufc2	0.001	0.022	0.040	0.000	-0.180	0.890	0.650	0.920
Q91VD9	Ndufs1	0.002	0.025	0.000	0.200	-0.070	0.850	0.650	0.790
Q91WD5	Ndufs2	0.006	0.045	0.000	0.410	-0.120	0.950	0.910	1.040
Q9DCT2	Ndufs3	0.021	0.090	-0.340	0.080	0.000	0.600	0.510	1.070
Q9CXZ1	Ndufs4	0.012	0.067	-0.110	0.320	0.000	0.880	0.660	1.120
P52503	Ndufs6	0.095	0.237	-0.330	0.000	0.740	0.970	0.630	1.040
Q9DC70	Ndufs7	0.005	0.040	0.000	0.110	-0.180	0.680	0.470	0.570
Q8K3J1	Ndufs8	0.002	0.025	0.000	0.080	-0.030	0.750	0.500	0.550
Q91YT0	Ndufv1	0.001	0.019	0.000	0.030	-0.080	0.820	0.550	0.690
Q9D6J6	Ndufv2	0.049	0.154	0.000	0.340	-0.180	0.720	0.360	0.860
Q8BUY5	Timmdc1	0.012	0.067	0.000	0.620	-0.100	1.080	1.290	1.470
Q9D8Y1	Tmem126a	0.000	0.011	0.000	0.010	-0.060	1.380	1.100	1.350
Q9D1R1	Tmem126b	0.441	0.698	0.150	-0.370	0.000	-0.570	0.780	0.800
Q921N7	Tmem70	0.029	0.111	-0.290	3.890	0.000	5.300	5.780	6.340
Q8K2B3	Sdha	0.017	0.081	0.000	0.000	-0.200	0.720	0.300	0.450
Q9CQA3	Sdhb	0.005	0.042	0.000	0.130	-0.140	0.650	0.430	0.550
Q9D0M3	Cyc1	0.192	0.384	0.240	0.000	-0.040	0.020	0.590	0.600
Q9D855	Uqcrb	0.013	0.069	0.000	0.170	-0.230	0.670	0.450	0.550
Q9CZ13	Uqcrc1	0.015	0.076	0.080	0.000	-0.050	0.660	0.290	0.460
Q9DB77	Uqcrc2	0.140	0.306	0.000	0.130	-0.100	0.560	0.050	0.310
Q9CR68	Uqcrfs1	0.007	0.048	0.000	0.070	-0.110	0.660	0.350	0.520
Q9CQ69	Uqcrq	0.004	0.038	0.000	0.170	-0.020	0.640	0.470	0.460
Q9D2R6	Coa3	0.017	0.080	0.000	0.300	-0.250	1.120	0.610	0.900
Q8BJ03	Cox15	0.015	0.075	0.000	-0.640	0.350	1.240	1.100	1.810
P19783	Cox4i1	0.004	0.038	0.100	-0.020	0.000	0.710	0.420	0.700
P12787	Cox5a	0.002	0.028	0.000	0.140	-0.030	0.630	0.450	0.630
P19536	Cox5b	0.287	0.512	-0.200	0.100	0.000	0.200	-0.050	0.220
P56391	Cox6b1	0.015	0.076	0.030	0.000	-0.080	0.410	0.170	0.410
Q9CPQ1	Cox6c	0.008	0.052	0.000	0.150	-0.100	0.360	0.540	0.500
P48771	Cox7a2	0.957	1.000	-0.460	0.000	0.310	0.090	-0.410	0.130

Q62425	Ndufa4	0.219	0.419	-0.210	0.210	0.000	0.200	0.070	0.540
P09925	Surf1	0.000	0.013	0.070	0.000	-0.070	0.680	0.590	0.720
Q03265	Atp5a1	0.002	0.025	0.000	0.050	-0.110	0.800	0.540	0.810
P56480	Atp5b	0.009	0.057	0.000	0.260	-0.160	0.720	0.570	0.810
Q91VR2	Atp5c1	0.035	0.125	0.130	0.000	-0.030	1.150	0.540	0.470
Q9D3D9	Atp5d	0.126	0.287	-0.220	0.090	0.000	0.570	0.060	0.270
P56382	Atp5e	0.000	0.013	0.000	0.120	-0.080	0.910	0.750	0.860
Q9DCX2	Atp5h	0.025	0.101	0.000	0.470	-0.340	1.070	1.120	0.720
P97450	Atp5j	0.033	0.121	0.000	0.130	-0.630	1.010	0.420	1.140
P56135	Atp5j2	0.005	0.040	0.000	0.390	-0.090	0.920	1.130	0.970
Q9CPQ8	Atp5l	0.001	0.022	-0.230	0.080	0.000	0.680	0.760	0.800
Q9DB20	Atp5o	0.001	0.019	0.000	0.130	-0.070	0.900	0.670	0.900
O35143	Atpif1	0.013	0.069	0.260	0.000	-0.320	1.590	1.150	0.810
Q921N7	Tmem70	0.029	0.111	-0.290	3.890	0.000	5.300	5.780	6.340

Supp. Table 3. Proteins related to ETC were detected in CD4$^+$ T cells isolated from old mice after mito-transfer and in non-manipulated CD4$^+$ T cells from old mice. CD4$^+$ T cells from young and old mice, and from old mice after mito-transfer were cultured for 4 h before processing for mass spectrometry analysis. Data expressed as median protein Log2 fold change of CD4$^+$ T cells from old mice, from 3 individual old mice (paired experiment)

Uniprot ID	Gene ID	P.Val	FDR	O1	O2	O3	OM1	OM2	OM3
Q9R1C7	Prpf40a	0.057	0.169	0.000	0.036	-0.175	-0.196	-0.679	-0.769
Q9ES52	Inpp5d	0.069	0.191	-0.054	0.183	0.000	-0.248	-0.967	-0.380
Q9ERK4	Cse1l	0.041	0.138	0.073	-0.029	0.000	-0.350	-1.430	-0.960
Q9DBG6	Rpn2	0.000	0.012	0.000	0.145	-0.216	1.580	1.360	1.520
Q9D358	Acp1	0.010	0.060	0.000	0.034	-0.377	-0.763	-1.300	-1.250
Q9CX99	Grap	0.077	0.207	-0.922	0.152	0.000	-1.990	-2.090	-0.687
Q99L45	Eif2s2	0.540	0.807	0.342	-0.259	0.000	0.266	-0.631	-0.179
Q99JR1	Sfxn1	0.018	0.083	0.000	0.180	-0.179	0.403	0.408	0.393
Q99JF8	Psip1	0.090	0.230	-0.095	0.000	0.152	-0.474	-0.546	-0.054
Q921F2	Tardbp	0.080	0.211	0.004	0.000	-0.021	-0.142	-0.728	-0.349
Q8QZY9	Sf3b4	0.531	0.798	-2.010	0.478	0.000	-3.370	-0.510	-0.237
Q8K4I3	Arhgef6	0.010	0.058	-0.259	0.000	0.183	-0.812	-1.510	-1.100

Q8K2Z4	Ncapd2	0.437	0.694	0.059	-0.356	0.000	0.217	-0.607	-0.761
Q8K1I7	Wipf1	0.146	0.315	-0.001	0.000	0.402	-0.311	-0.419	0.062
Q8CC88	Vwa8	0.942	1.000	0.000	0.045	-0.313	0.847	-0.752	-0.481
Q8C3J5	Dock2	0.006	0.047	0.009	0.000	-0.011	-0.628	-1.100	-0.665
Q8C2K5	Rasal3	0.018	0.083	-0.082	0.141	0.000	-0.512	-1.320	-0.924
Q8BZN6	Dock10	0.210	0.406	-0.532	0.000	0.228	-0.481	-1.600	-0.303
Q8BK67	Rcc2	0.253	0.466	0.487	-0.092	0.000	0.079	-0.473	-0.172
Q8BH59	Slc25a12	0.037	0.127	-0.084	0.153	0.000	0.368	0.204	0.480
Q8BGW0	Themis	0.127	0.288	-1.320	0.000	0.443	-1.890	-1.260	-1.100
Q80SU7	Gvin1	0.117	0.273	0.231	0.000	-0.019	0.045	-0.881	-0.733
Q78ZA7	Nap1l4	0.009	0.056	-0.293	0.000	0.003	-0.919	-1.530	-0.967
Q6ZQ38	Cand1	0.009	0.057	-0.021	0.028	0.000	-0.389	-0.813	-0.557
Q6PDI5	Ecm29	0.133	0.297	0.000	-0.153	0.001	0.008	-1.570	-1.600
Q6PB66	Lrpprc	0.035	0.125	0.000	0.121	-0.292	0.941	0.428	0.430
Q62351	Tfrc	0.848	1.000	1.330	0.000	-0.491	1.130	-0.397	-0.350
Q62077	Plcg1	0.003	0.033	-0.125	0.000	0.471	-1.190	-1.370	-1.750
Q61823	Pdcd4	0.011	0.065	-0.098	0.000	0.177	-1.090	-2.030	-1.100
Q61081	Cdc37	0.002	0.027	0.019	0.000	-0.009	-0.837	-1.370	-1.050
Q60932	Vdac1	0.006	0.046	0.000	0.313	-0.052	1.240	1.010	1.700
Q60931	Vdac3	0.007	0.050	0.000	0.252	-0.058	1.080	0.902	1.570
Q60787	Lcp2	0.009	0.057	-0.096	0.845	0.000	-1.390	-1.550	-1.040
Q60631	Grb2	0.005	0.041	-0.091	0.403	0.000	-1.080	-1.510	-0.944
Q3UUV5	Skap1	0.002	0.023	-0.314	0.000	0.236	-1.930	-2.300	-1.630
Q3UPF5	Zc3hav1	0.070	0.193	-0.342	0.000	0.012	-0.376	-0.579	-0.367
Q3UND0	Skap2	0.206	0.403	0.518	0.000	-0.624	-0.258	-0.599	-1.130
Q3TBT3	Tmem173	0.649	0.925	-0.395	0.164	0.000	-0.291	-0.323	0.074
Q1HFZ0	Nsun2	0.102	0.247	0.289	0.000	-0.086	-0.029	-1.110	-0.849
Q03526	Itk	0.005	0.041	0.620	-0.355	0.000	-2.150	-1.480	-1.840
Q01965	Ly9	0.539	0.807	0.248	-0.106	0.000	-0.213	-0.306	0.254
P97370	Atp1b3	0.009	0.056	0.000	0.096	-0.048	0.414	0.419	0.703
P70218	Map4k1	0.001	0.015	-0.001	0.000	0.020	-0.901	-1.290	-1.110
P70168	Kpnb1	0.204	0.400	-0.040	0.088	0.000	-0.081	-0.545	-0.064
P68254	Ywhaq	0.006	0.046	-0.139	0.000	0.050	-0.858	-1.630	-1.230
P63101	Ywhaz	0.001	0.015	0.000	-0.021	0.113	-1.140	-1.610	-1.370
P63037	Dnaja1	0.340	0.580	0.072	0.000	-0.107	0.131	-0.492	-0.294
P62259	Ywhae	0.027	0.105	0.000	0.068	-0.183	-0.420	-1.160	-0.884
P61982	Ywhag	0.016	0.078	0.076	0.000	-0.065	-0.399	-0.977	-0.678
P61290	Psme3	0.001	0.021	0.000	0.420	-0.049	-1.120	-1.150	-1.340
P54775	Psmc4	0.018	0.083	-0.123	0.000	0.106	-0.391	-0.884	-0.580
P49718	Mcm5	0.432	0.688	0.140	0.000	-0.032	0.135	-0.802	-0.001
P43404	Zap70	0.038	0.131	-0.465	0.041	0.000	-0.669	-0.946	-0.589
P42227	Stat3	0.103	0.250	0.000	-0.155	0.131	-0.089	-0.977	-0.688

P42225	Stat1	0.005	0.039	-0.079	0.271	0.000	-0.603	-0.992	-0.866
P39688	Fyn	0.582	0.853	-0.633	0.103	0.000	-0.287	-0.529	-0.169
P39054	Dnm2	0.326	0.562	0.075	-0.001	0.000	0.448	-0.098	0.271
P36371	Tap2	0.787	1.000	-0.225	0.273	0.000	-0.153	-0.078	0.133
P27870	Vav1	0.049	0.154	0.325	0.000	-0.114	-0.272	-1.240	-1.350
P26450	Pik3r1	0.468	0.727	0.000	0.958	-0.100	-0.021	0.030	0.038
P26039	Tln1	0.041	0.137	0.059	0.000	-0.282	-0.358	-1.020	-1.000
P25206	Mcm3	0.066	0.185	0.280	0.000	-0.196	-0.205	-1.110	-0.943
P24161	Cd247	0.811	1.000	-0.617	0.000	0.247	-0.627	0.147	0.420
P24063	Itgal	0.054	0.163	-0.246	0.010	0.000	-0.641	-0.514	-0.247
P22682	Cbl	0.000	0.013	-0.024	0.000	0.154	-1.190	-1.550	-1.380
P22646	Cd3e	0.501	0.766	-0.349	0.000	0.371	-0.475	-0.277	0.156
P19783	Cox4i1	0.004	0.038	0.100	-0.024	0.000	0.711	0.422	0.696
P11942	Cd3g	0.167	0.349	-0.367	0.000	0.062	-0.670	-0.495	-0.158
P11352	Gpx1	0.131	0.294	0.457	0.000	-0.235	-0.028	-0.804	-0.877
P08113	Hsp90b1	0.004	0.035	0.176	0.000	-0.151	1.090	0.696	0.949
P04235	Cd3d	0.281	0.504	-0.353	0.097	0.000	-0.435	-0.393	-0.084
P01851	Tcb2	0.061	0.176	-0.290	0.000	0.247	-0.718	-0.420	-0.372
O89100	Grap2	0.049	0.154	-0.257	0.110	0.000	-0.559	-2.360	-1.760
O54957	Lat	0.507	0.771	-0.255	0.567	0.000	0.081	0.134	0.862
O54734	Ddost	0.002	0.025	0.000	0.042	-0.469	1.490	1.120	1.220

Supp. Table 4. Proteins related to TCR signalosome were detected in $CD4^+$ T cells isolated from old mice after mito-transfer and in non-manipulated $CD4^+$ T cells from old mice. $CD4^+$ T cells from young and old mice, and from old mice after mito-transfer were cultured for 4 h before processing for mass spectrometry analysis. Data expressed as median protein Log2 fold change of $CD4^+$ T cells from old mice, from 3 individual old mice (paired experiment)
